# Evaluation of the Correlation between Serum Concentrations of Asymmetric Dimethylarginine and Corrected TIMI Frame Count in Patients with Slow Coronary Flow

**DOI:** 10.1155/2020/4592190

**Published:** 2020-09-19

**Authors:** Mahshid Naserifar, Mahshid Ataei, Nadia Behzadian, Amir Hooshang Mohammadpour, Mostafa Dastani, Amirhossein Sahebkar, Hesamoddin Hosseinjani

**Affiliations:** ^1^Department of Clinical Pharmacy, School of Pharmacy, Mashhad University of Medical Sciences, Mashhad, Iran; ^2^Pharmaceutical Research Center, Pharmaceutical Technology Institute, Mashhad University of Medical Sciences, Mashhad, Iran; ^3^Department of Cardiology, School of Medicine, Mashhad University of Medical Sciences, Mashhad, Iran; ^4^Biotechnology Research Center, Pharmaceutical Technology Institute, Mashhad University of Medical Sciences, Mashhad, Iran; ^5^Neurogenic Inflammation Research Center, Mashhad University of Medical Sciences, Mashhad, Iran; ^6^Polish Mother's Memorial Hospital Research Institute (PMMHRI), Lodz, Poland

## Abstract

Coronary slow flow (CSF) is an important angiographic entity that is characterized by delayed opacification of coronary arteries in the absence of epicardial occlusive disease. Asymmetric dimethylarginine (ADMA) is an endogenous inhibitor of nitric oxide synthase. Elevated levels of ADMA cause the induction of endothelial dysfunction and thus promote atherosclerosis. This study was aimed at determining the role of ADMA in the development of CSF. One hundred twenty-nine subjects who fulfilled the inclusion criteria were enrolled in this study. According to coronary angiography results, these subjects were divided into five groups. The serum concentration of ADMA was measured in these subjects. In this study, there was no significant correlation between serum concentrations of ADMA and mean corrected TIMI frame count (CTFC) (*P* > 0.05). However, the ADMA level was significantly correlated with CTFC in the left anterior descending (LAD) coronary artery in patients with CSF (*r* = −0.381, *P* = 0.045). Also, plasma ADMA levels were significantly higher in patients with CSF and without CAD compared to patients without CSF and with CAD (50-90%) (*P* = 0.034). Besides, serum concentrations of ADMA were significantly higher in subjects with BMI < 25 kg/m^2^ compared with those having BMI > 30 kg/m^2^ (*P* = 0.003). It was also shown that the levels of ADMA were significantly higher in subjects with age as a cardiovascular risk factor compared with those without this risk factor (*P* = 0.024). Further studies with larger population sizes are needed to confirm the present findings on the association between the serum concentrations of ADMA and CSF.

## 1. Introduction

Patients who have chest pain without coronary artery disease (CAD) present a diagnostic and therapeutic challenge and impose high costs on society and the medical system. At least 10-30% of patients with angina lack significant CAD in coronary angiography. 50-65% of these patients, who have chest pain without obstructive CAD, have coronary microvascular disorders, often known as microvascular angina. There are various diagnoses to justify chest pain without obstructive CAD which include microvascular angina, gastroesophageal reflux, musculoskeletal chest pain, cardiac syndrome X, coronary artery slow flow, coronary vasospasm, and lack of coronary artery reperfusion [1].

Coronary slow flow phenomenon (CSFP) is an angiographic finding that is diagnosed by delayed terminal vascular filling in the absence of significant epicardial CAD [2]. However, the exact etiology is still unknown [3]. This phenomenon is clinically quite debilitating. It is diagnosed through angiographic findings of difference in corrected thrombolysis in myocardial infarction (TIMI) frame count of more than two standard deviations of the normal range in the absence of coronary artery obstruction [4]. Corrected TIMI frame count (CTFC) is a quantitative and repeatable index of coronary artery flow. CTFC shows the number of frames needed for the contrast agents to achieve the standardized terminal coronary artery. The normal TIMI frame count of the LAD vessel (36.2 ± 2.6) is 1.7 times longer than the left circumflex artery (LCX) (22.4 ± 2.1) and the right coronary artery (RCA) (20.3 ± 4) vessels [2].

The discussed etiologies of this phenomenon include small vessel disease, microvascular vasomotor disorder, diffuse atherosclerosis, and endothelial dysfunction. Morphological and functional abnormalities are involved in the pathogenesis of CSFP in small vessels and epicardial coronary arteries [4]. Articles have suggested that endothelial dysfunction plays an important pathogenic role in patients with CSFP [5].

ADMA is an intracellular amino acid produced by methyltransferases during arginine methylation. In physiological conditions, ADMA is present in plasma, urine, tissues, and cells. ADMA is a competitive endogenous nitric oxide synthase inhibitor [6]. In vivo studies have shown that increased ADMA significantly inhibits nitric oxide synthase and decreases nitric oxide production in endothelial cells and human blood vessels [7]. ADMA values are associated with peripheral atherosclerosis and the incidence of cardiovascular events in patients with CAD [8]. Minor changes in plasma ADMA levels can cause significant changes in the intracellular level of this compound, which can cause alteration in nitric oxide production and advancing cardiovascular disease. In pathological conditions, ADMA can increase 3- to 9-fold, which significantly increases the inhibition of nitric oxide production [7].

Coronary artery slow flow is a phenomenon that still lacks sufficient information on its cause and how it occurs. Given that the pathophysiology of this disease includes microvascular disorders, endothelial dysfunction, subclinical atherosclerosis, inflammation, and anatomic factors, diagnosing the causes of these microvascular disorders and endothelial dysfunctions and how they occur is essential to understand this phenomenon better. On the other hand, the role of ADMA in the occurrence of microvascular diseases and endothelial dysfunction has been proved in previous studies, and the mechanism of these disorders has been investigated.

However, few studies have examined the role of this biomarker in CSFP and its occurrence. It seems that investigating this issue could help finding better methods for treating patients with CSFP and increase recognition of this phenomenon and its involved mechanisms. Considering the limited number of studies to evaluate ADMA as a biomarker in patients with CSFP, it seems necessary to conduct a study to examine the role of this biomarker in CSFP. Currently, angiography is the only way to detect slow flow of coronary arteries and follow-up treatment in patients with CSFP, but it is an invasive procedure. Therefore, this studyaimed to find a correlation between the serum concentration of ADMA and CSFP which can help in the diagnosis and treatment follow-up of patients without the need for an invasive procedure. Also, we aimed to compare serum concentrations of ADMA between patient groups with and without cardiovascular risk factors to the potential role of ADMA as a cardiovascular risk marker.

## 2. Patients and Methods

Adult patients referred for coronary angiography to Ghaem Hospital of Mashhad University of Medical Sciences were evaluated for inclusion and exclusion criteria to participate in the study. The inclusion criterion was candidates for coronary angiography, and the exclusion criteria included dialysis patients, coronary artery aneurysm, hyperhomocysteinemia, myocarditis, pericarditis, cardiomyopathy, and using sitagliptin. A total number of 129 patients who underwent angiography were entered into this pilot study. Written informed consent was obtained from all patients.

Based on angiographic results, patients were divided into five groups:

1. Patients without CAD and epicardial CSF
2. Patients without CAD and with epicardial CSF
3. Patients with CAD (less than 50%) and without epicardial CSF
4. Patients with CAD (50-90%) and without epicardial CSF
5. Patients with CAD and epicardial CSF

Demographic information such as age and sex, history of chronic diseases, cigarette smoking, family history of heart disorders, and patients' angiographic results were filled individually in the designed forms. A blood sample of 20 mL was drawn from the brachial vein of each patient and centrifuged at 3000 rpm in the Emergency Laboratory of Ghaem Hospital. After centrifugation, the samples were kept in a -80°C freezer for laboratory analysis.

After the end of the sampling phase, samples were taken from the freezer to measure the level of ADMA using ELISA Kit according to a standard protocol, including preparation of reactants and samples for the assay process.

## 3. Statistical Method

Descriptive statistics included the mean and standard deviation for quantitative variables with normal distribution, median and interquartile range for quantitative variables with nonnormal distribution, and frequency percentage for qualitative variables. For statistical analysis, the normality of the distribution of quantitative variables was evaluated using the Kolmogorov-Smirnov test. In the case of a normal distribution of variables, independent *t*-test and variance analysis were used to compare the mean of quantitative variables between two and more than two independent groups, respectively. In the absence of normal distribution, nonparametric tests such as Mann-Whitney *U* test and Kruskal-Wallis test were used to compare the quantitative variables between two and more than two independent groups, respectively. It is noteworthy that in the case of rejecting the hypothesis of equality of means in the analysis of variance, the Tukey multiple comparison test was used to find out which difference between pairs of means caused a significant difference. Pearson correlation coefficient (if normal) and Spearman correlation coefficient (if non-normal) were used to investigate the severity and direction of the relationship between two variables. In all tests, a two-sided *P* value of <0.05 was considered statistically significant.

## 4. Results

A total of 129 patients who had undergone angiography entered the study. Of these, 51 (39.53%) were male, and the mean age of the study population was 54.27 ± 10.30 years.

### 4.1. Frequency Distribution of Patients in terms of Vascular Congestion Status and Epicardial CSF

Patients without CAD and epicardial CSF had the highest frequency (31%), followed by patients with less than 50% CAD and without epicardial CSF (22.48%). The frequency percentages of patients without CAD and with epicardial CSF and patients with more than 50% and less than 90% CAD without epicardial CSF were both 17.05%. Finally, patients with CAD and epicardial CSF had the least frequency between groups (12.40%).

### 4.2. Frequency Distribution of the Number of Vessels Involved in Patients with CAD

A total of 22 patients had CAD between 50 and 90%. The highest frequency of the number of vessels involved in these patients was related to one coronary artery involvement (36.36% of cases). Also, the frequency percentages of patients with two or three coronary vessel involvement were both 31.81%.

### 4.3. Comparison of the Mean Serum Concentration of ADMA in Study Groups

Table 1 shows the mean and standard deviation of the serum level of ADMA in different groups of patients. As can be observed, the mean serum level of ADMA had a significant difference between groups (*P* = 0.034).

The post hoc test was done to find out which differences between the two mean serum levels of ADMA among five study groups were significant. The results of paired comparison of the mean ADMA serum concentration in study groups with ANOVA statistical test showed that the serum level of ADMA in patients without CAD and with epicardial CSF was significantly different from those with CAD (50-90%) and without epicardial CSF (*P* = 0.034). However, the comparison between other groups showed no significant difference.

### 4.4. Comparison of the Mean Serum Concentration of ADMA in Patients with or without Cardiac Risk Factors

Table 2 indicates the mean and standard deviation of the serum concentration of ADMA in patients with or without hypertension, diabetes mellitus, dyslipidemia, age risk factor, or smoking. As can be observed, the mean serum concentration of ADMA had no significant difference among patients with or without hypertension, diabetes mellitus, dyslipidemia, or smoking. Age over 45 and 55 years in male and female subjects, respectively, was considered a cardiovascular risk factor.

As can be seen in Table 2, the two groups were significantly different in the mean serum concentration of ADMA (*P* = 0.024). So the serum concentration of ADMA in patients with age risk factors was significantly lower than that in the other patient groups.

### 4.5. Comparison of the Mean Serum Concentration of ADMA in Patient Groups Based on BMI

Table 3 shows the mean and standard deviation of the serum concentration of ADMA in patient groups with different BMI ranges. As can be seen, the mean serum concentration of ADMA had a significant difference among patients with different BMI ranges.

Post-hoc test was used to explore the difference in serum levels of ADMA in group pairs. The results of the one-way ANOVA test indicated that the mean serum concentration of ADMA in the group with a BMI less than 25 was significantly more than that in the group with a BMI more than 30 (*P* = 0.003). The comparison between other groups did not show any significant differences.

### 4.6. Relationship between the Serum Concentration of ADMA and CTFC-Based Coronary Artery Slow Flow Intensity

As shown in Table 4, the serum concentration of ADMA was not significantly correlated with coronary artery slow flow intensity based on mean CTFC in different vessels. However, the serum level of ADMA had a significant inverse relationship with coronary artery slow flow intensity based on CTFC in the LAD vessel (*P* = 0.045). Nevertheless, there was no significant relationship between the serum level of ADMA and coronary artery slow flow intensity based on CTFC in the RCA and LCX vessels.

## 5. Discussion

CSFP is an important clinical, angiographic finding because it can result in angina during rest or exercise, acute myocardial infarction, and hypertension in some patients [9]. CSFP can be marked by delayed filling of the terminal vessels in the absence of epicardial coronary artery obstruction [2].

However, it is identified that factors like microvascular disorders, coronary endothelial dysfunctions, subclinical atherosclerosis, inflammation, and anatomic factors can be considered pathophysiological factors in this disease [5].

ADMA is a competitive nitric oxide synthase inhibitor, which is associated with peripheral atherosclerosis and cardiovascular events in patients with CAD [6, 8].

### 5.1. Main Finding

The present study has evaluated the ADMA level in patients with or without CAD and also with or without CSF. The mean serum concentration did not show any significant correlation with the intensity of CSF based on mean CTFC in different vessels. Given the role of this biomarker in the development and progression of endothelial dysfunction in previous studies, it may be argued that the cause of the inconsistency in the results may be the important role of other factors such as inflammation in the pathogenesis of CSF. In this study, the mean serum concentration of ADMA in LAD vessels had a significant inverse correlation with the intensity of CSF based on CTFC. Besides, the mean serum concentration of ADMA in patients without CAD and with epicardial CSF was significantly more than that in patients with CAD (50-90%) and without CSF. There was also a significant difference in the mean serum concentration of ADMA between groups according to the age risk factor and BMI in a group with BMI < 25 and another group with BMI > 30.

### 5.2. Comparison with Similar Studies

In a study conducted in 2012 on 40 patients with CSF and 23 people as a control group, no significant difference was observed in the plasma concentration of ADMA [10]. In a study which was done in 2007 on 62 persons (31 with CSF and 31 with normal coronary blood flow) in which coronary flow was measured using the TIMI frame count method, plasma concentrations of ADMA in patients with CSF were significantly higher in comparison to those in the healthy group (*P* = 0.006). Also, the ratio of L-arginine/ADMA was significantly lower in a group with CSF compared to the healthy group (*P* = 0.002). In this study, ADMA concentration had a significant correlation with the mean TIMI frame count in different vessels and also with the TIMI frame count in each vessel in patients with CSF [11]. However, in the present study, the serum concentration of ADMA was only significantly correlated with the TIMI frame count in the LAD vessel.

Also, in a study conducted in 2011 on 50 patients with CSF and 30 persons with normal coronary arteries and normal coronary blood flow, the mean TIMI frame count was significantly correlated with the plasma concentration of ADMA (*r* = 0.26, *P* = 0.02). In this study, also, the serum concentration of ADMA in a group with CSF was significantly more than that in the other group with the normal flow [12]. Similarly, the results of the present study demonstrated that in one group with CSF, the serum concentration of ADMA is significantly higher than that in another group without CSF.

In a study done by Perticone et al. in 2008 on 84 people (21 with normal blood pressure and 63 with hypertension), plasma concentrations of ADMA and L-arginine were significantly higher in patients with hypertension than in the other group with normal blood pressure (the mean plasma concentration of ADMA in a group with normal blood pressure was 0.4 ± 0.1 *μ*mol/L and in a group with hypertension was 0.6 ± 0.1 *μ*mol/L, *P* = 0.0001) [13]. However, no such correlation was observed in our study.

Resistance to insulin is a condition that may enhance the incidence and spread of atherosclerosis due to its association with endothelial dysfunction, vasoconstriction, inflammation, and apoptosis [13]. In a 2016 study of 601 patients, serum concentrations of ADMA were significantly higher in diabetic patients (0.505 ± 0.112 *μ*mol/L) than in the nondiabetic group (0.458 ± 0.104 *μ*mol/L) (*P* = 0/0003) [14]. However, in the present study, no such correlation was observed, which could be due to the smaller sample size than the mentioned study.

Metabolic syndrome is a group of pathophysiological changes that include hypertension, insulin resistance, dyslipidemia, and abdominal obesity. In a 2010 study by Palermo et al., on 85 people, patients were divided into two groups, 48 people with metabolic syndrome and 37 people without metabolic syndrome as a control group. The ADMA value was significantly higher in a group with metabolic syndrome (0.71 ± 0.38) than the group without metabolic syndrome (0.48 ± 0.28) (*P* = 0.0009). In this study, ADMA values were significantly correlated with waist circumference (*P* = 0.01) but not with other components of metabolic syndrome. These results suggest a possible association between elevated levels of ADMA and metabolic syndrome [15].

Obesity is a significant risk factor for the development of type 2 diabetes and cardiovascular disease [16]. In a 2010 study on 29 people (17 lean and 12 obese), obesity or fat intake had no significant effect on ADMA plasma concentration. In this study, the mean plasma concentration of ADMA in the lean group was 0.51 *μ*mol/L after a low-fat diet and 0.52 *μ*mol/L after a high-fat diet. Also, the mean plasma concentration of ADMA in the obese group after a low- and a high-fat diet was both 0.53 *μ*mol/L [17]. However, in the present study, the mean plasma ADMA concentration had a significant difference between the two groups with BMI less than 25 and those with BMI more than 30 in a way that the mean plasma ADMA concentration in a group with BMI less than 25 was higher than that in the other group.

Another risk factor examined in this study was smoking. Smoking is a major risk factor for atherosclerosis, brain and cardiovascular disease, hypertension, and diabetes mellitus. In a 2016 study on 601 patients, smoker patients had a higher ADMA serum concentration (0.532 ± 0.142 *μ*mol/L) than nonsmokers (0.468 ± 0.107 *μ*mol/L) (*P* = 0.001) [14]. However, there was no difference in the present study, which may be due to the smaller sample size than the study mentioned above.

In our study, the effect of age as an established cardiovascular risk factor was also examined. The mean serum ADMA concentration had a significant difference between the two groups with and without the age risk factor. Accordingly, the mean serum level of this biomarker in a group without the age risk factor was higher than that in the other group. However, other studies have not examined this risk factor.

Some of the limitations of the present study were the relatively small sample size and lack of follow-up for long-term events and outcomes. The present study was the first to investigate the correlation between epicardial CSF and ADMA level in which patients were divided into five groups as one of the strengths of this study. The results of the present study indicated the role of ADMA in cardiovascular diseases as well as its association with some related risk factors. However, further studies with larger sample sizes are needed to confirm the present results and determine this relationship.

## Figures and Tables

**Table 1:** Comparison of the mean serum concentration of ADMA in study groups.

Group	Serum level of ADMA (mean ± standard deviation)
CAD (-), slow flow (-)	0.29 ± 0.13
CAD (-), slow flow (+)	0.32 ± 0.14
CAD (+) (<50%), slow flow (-)	0.30 ± 0.18
CAD (+) (50-90%), slow flow (-)	0.20 ± 0.07
CAD (+), slow flow (+)	0.25 ± 0.14

CAD: coronary artery disease.

**Table 2:** Comparison of the serum concentration of ADMA in patients with or without cardiac risk factors.

Group	Serum level of ADMA (mean ± standard deviation)	*P* value^∗^
With hypertension	0.28 ± 0.15	0.672
Without hypertension	0.26 ± 0.13	
With diabetes mellitus	0.29 ± 0.16	0.422
Without diabetes mellitus	0.27 ± 0.13	
With dyslipidemia	0.28 ± 0.14	0.591
Without dyslipidemia	0.27 ± 0.14	
With age risk factor	0.25 ± 0.13	0.024
Without age risk factor	0.31 ± 0.15	
With smoking	0.29 ± 0.14	0.535
Without smoking	0.27 ± 0.14	

^∗^Independent-sample *t*-test.

**Table 3:** Comparison of the mean serum concentration of ADMA in patient groups with different BMI ranges.

Body mass index (kg/m^2^)	Serum level of ADMA (mean ± standard deviation)
Less than 25	0.35 ± 0.15
Between 25 and 26.9	0.29 ± 0.13
Between 27 and 29.9	0.26 ± 0.14
More than 30	0.23 ± 0.18

**Table 4:** Relationship between the serum concentration of ADMA and coronary artery slow flow intensity based on CTFC.

Variable	Correlation coefficient	*P* value∗
Mean CTFC in different vessels	0.25	0.140
CTFC in LAD vessel	0.38	0.045
CTFC in RCA vessel	0.41	0.088
CTFC in LCX vessel	0.38	0.152

^∗^Pearson statistical test.

## Data Availability

The data used to support the findings of this study are available from the corresponding author upon request.
