# Corrigendum to “Evaluation of the Correlation between Serum Concentrations of Asymmetric Dimethylarginine and Corrected TIMI Frame Count in Patients with Slow Coronary Flow”

**DOI:** 10.1155/2020/9796012

**Published:** 2020-12-11

**Authors:** Mahshid Naserifar, Mahshid Ataei, Nadia Behzadian, Amir Hooshang Mohammadpour, Mostafa Dastani, Jamal Shamsara, Sepideh Elyasi, Amirhossein Sahebkar, Hesamoddin Hosseinjani

**Affiliations:** ^1^Department of Clinical Pharmacy, School of Pharmacy, Mashhad University of Medical Sciences, Mashhad, Iran; ^2^Pharmaceutical Research Center, Pharmaceutical Technology Institute, Mashhad University of Medical Sciences, Mashhad, Iran; ^3^Department of Cardiology, School of Medicine, Mashhad University of Medical Sciences, Mashhad, Iran; ^4^Biotechnology Research Center, Pharmaceutical Technology Institute, Mashhad University of Medical Sciences, Mashhad, Iran; ^5^Neurogenic Inflammation Research Center, Mashhad University of Medical Sciences, Mashhad, Iran; ^6^Polish Mother's Memorial Hospital Research Institute (PMMHRI), Lodz, Poland

In the article titled “Evaluation of the Correlation between Serum Concentrations of Asymmetric Dimethylarginine and Corrected TIMI Frame Count in Patients with Slow Coronary Flow” [1], Dr. Jamal Shamsara and Dr. Sepideh Elyasi were missing from the author list. Dr. Elyasi contributed to the study planning, data analysis, and manuscript preparation. Dr. Shamsara contributed to the study design, data analysis, and manuscript preparation. The corrected author list is shown above.
